# Model-based evaluation of the long-term cost-effectiveness of systematic case-finding for COPD in primary care

**DOI:** 10.1136/thoraxjnl-2018-212148

**Published:** 2019-07-08

**Authors:** Tosin Lambe, Peymane Adab, Rachel E Jordan, Alice Sitch, Alex Enocson, Kate Jolly, Jen Marsh, Richard Riley, Martin Miller, Brendan G Cooper, Alice Margaret Turner, Jon G Ayres, Robert Stockley, Sheila Greenfield, Stanley Siebert, Amanda Daley, KK Cheng, David Fitzmaurice, Sue Jowett

**Affiliations:** 1 Health Economics Unit, University of Birmingham, Birmingham, UK; 2 Institute of Applied Health Research, The University of Birmingham, Birmingham, UK; 3 Research Institute for Primary Care and Health Sciences, Keele University, Staffordshire, UK; 4 Institute of Occupational and Environmental Medicine, University of Birmingham, Birmingham, West Midlands, UK; 5 Institute of Applied Health Research, University of Birmingham, Birmingham, West Midlands, UK; 6 Lung Investigation Unit, University Hospital Birmingham, Birmingham, UK; 7 School of Clinical and Experimental Medicine, University of Birmingham, Birmingham, Birmingham, UK; 8 Institute of Occupational and Environmental Med, University of Birmingham, Birmingham, UK; 9 Queen Elizabeth Hospital, Birmingham, UK; 10 Birmingham Business School, University of Birmingham, Birmingham, UK

**Keywords:** COPD, markov model, case-finding, early diagnosis, cost-effectiveness

## Abstract

**Introduction:**

‘One-off’ systematic case-finding for COPD using a respiratory screening questionnaire is more effective and cost-effective than routine care at identifying new cases. However, it is not known whether early diagnosis and treatment is beneficial in the longer term. We estimated the long-term cost-effectiveness of a regular case-finding programme in primary care.

**Methods:**

A Markov decision analytic model was developed to compare the cost-effectiveness of a 3-yearly systematic case-finding programme targeted to ever smokers aged ≥50 years with the current routine diagnostic process in UK primary care. Patient-level data on case-finding pathways was obtained from a large randomised controlled trial. Information on the natural history of COPD and treatment effects was obtained from a linked COPD cohort, UK primary care database and published literature. The discounted lifetime cost per quality-adjusted life-year (QALY) gained was calculated from a health service perspective.

**Results:**

The incremental cost-effectiveness ratio of systematic case-finding versus current care was £16 596 per additional QALY gained, with a 78% probability of cost-effectiveness at a £20 000 per QALY willingness-to-pay threshold. The base case result was robust to multiple one-way sensitivity analyses. The main drivers were response rate to the initial screening questionnaire and attendance rate for the confirmatory spirometry test.

**Discussion:**

Regular systematic case-finding for COPD using a screening questionnaire in primary care is likely to be cost-effective in the long-term despite uncertainties in treatment effectiveness. Further knowledge of the natural history of case-found patients and the effectiveness of their management will improve confidence to implement such an approach.

**Key messages**

**What is the key question?**

- What is the long-term cost-effectiveness of undertaking a regular programme of case-finding and early detection of COPD?

**What is the bottom line?**

- Health economic decision modelling found that systematic case-finding among ever-smokers aged 50 years and over on a 3-yearly basis is highly likely to be cost-effective compared with routine practice.

**Why read on?**

- Currently, case-finding programmes for COPD are not being implemented internationally due to the lack of evidence on the long-term benefits and cost-effectiveness of early diagnosis, and this paper presents the first analysis of the long-term cost-effectiveness of systematic case-finding for undiagnosed COPD.

## Introduction

COPD is one of the most common long-term conditions with significant public health impact, costing over £1.5 billion per annum to the UK National Health Service (NHS),^1^ largely due to emergency hospital admissions among patients experiencing exacerbations and costs of maintenance medication.^2^ Despite considerable health service use,^3^ it is thought that perhaps half of all subjects with this disease still remain undiagnosed.^4^ Smoking cessation interventions, pharmacotherapy and non-pharmacological approaches such as pulmonary rehabilitation and self-management can reduce morbidity, particularly the frequency of exacerbations and prolong the life of patients diagnosed of COPD.^5–9^ Observed benefits might be even greater if undiagnosed patients were found earlier and appropriate treatment commenced, although evidence to support this is currently limited.^10^

A number of small uncontrolled studies of different approaches to identify patients with undiagnosed COPD from primary care and other settings have been undertaken,^11^ but there are few appropriately designed trials to address this issue. We recently conducted the largest cluster randomised controlled trial (TargetCOPD)^12^ to evaluate two alternative systematic approaches to identify undiagnosed symptomatic patients compared with routine practice (no systematic case-finding). The systematic strategies consisted of opportunistic case-finding, where a respiratory screening questionnaire was administered when eligible patients attended their primary care practice for consultation, and an active approach, where patients were additionally invited by mail to complete the same questionnaire. In both cases, symptomatic patients were then invited for diagnostic spirometry. Over the 1-year trial period, active case-finding was the most effective and cost-effective approach to identify new cases (OR=7.5 (95% CI 4.80 to 11.55); £333 per additional case detected) compared with routine practice.

Although short-term clinical and cost-effectiveness of a single ‘one-off’ programme of case-finding was demonstrated, this does not necessarily translate into future long-term benefits for a regular programme. Furthermore, the results of the economic analysis (cost per case detected) are not easily comparable with results from other health programmes.^13^ In the absence of long-term trial data, model-based economic evaluations are needed.^14^ We report the results of a model-based economic evaluation of the long-term costs and benefits of a regular programme of systematic active case-finding over routine practice, using data from the TargetCOPD trial,^12^ the linked Birmingham COPD cohort,^15^ a large primary care database and the published literature. The model outcome is expressed in cost per quality-adjusted life-year (QALY) gained, a measure where a cost-effectiveness decision threshold rule exists in the UK.^16^

## Methods

### Study design and intervention

A Markov decision model was built with TreeAgePro 2015 (TreeAge Software, Williamstown, Massachusetts, USA) to estimate the long-term cost-effectiveness of systematic active case-finding for COPD among ever-smokers without a prior diagnosis of COPD in primary care versus routine practice. A cost–utility analysis was undertaken to calculate the cost per QALY gained from a health service perspective (the UK NHS).

The model was based on the methods in our published case-finding trial, using data from the most effective strategy identified in the trial (‘active’ case-finding) compared with routine care.^12 17^ The population in the trial comprised ever smokers aged 40–79 years without a prior diagnosis of COPD. However, for this long-term model, we chose a starting cohort of those aged 50 years, as few patients were identified below this age in the trial.^12^

For the active case-finding approach, eligible patients were identified through electronic health records using a standardised search and their records ‘flagged’. Flagged patients were offered a respiratory symptom screening questionnaire at any routine practice visit and were also sent the questionnaire by mail with a reply-paid envelope with up to two reminders. Patients who reported relevant chronic respiratory symptoms on the questionnaire were invited for a confirmatory spirometry test to diagnose COPD according to UK criteria.^18^ Routine practice was defined according to UK and international guidance,^19^ which recommends spirometric confirmation of COPD among those over the age of 35 years who have a risk factor (generally smoking) and who present with exertional breathlessness, chronic cough, regular sputum production, frequent winter ‘bronchitis’ or wheeze.^18^ Case-finding was a one-off activity in the TargetCOPD trial, but in this study, we have assumed that the intervention would be repeated every 3 years.

### Model structure

**Figure 1:**
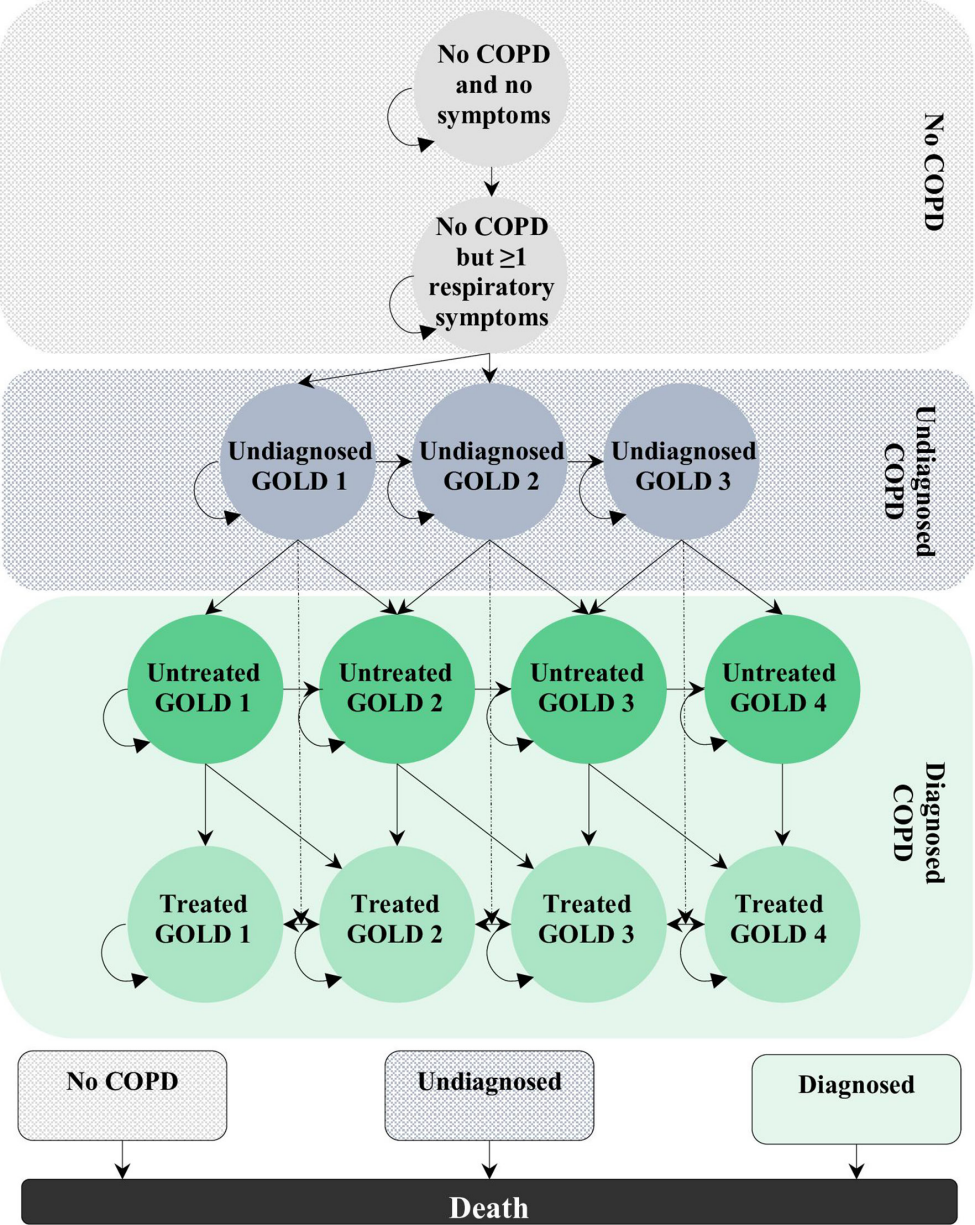
Transitions between model health states.

Patients without a prior COPD diagnosis in each strategy moved between 14 mutually exclusive health states over their lifetime (figure 1). The health states were grouped into three broad disease categories: disease free, undiagnosed disease, diagnosed disease and dead. Patients with no airflow obstruction, either with or without respiratory symptoms, were classified as ‘disease free’. Those with relevant respiratory symptoms and airflow obstruction were classified as either remaining undiagnosed or becoming diagnosed. A diagnosis required either a new health record of a COPD diagnosis through routine care or receiving a diagnosis through the case-finding programme.^18 20^ COPD health states were defined according to the traditional Global Initiative for Chronic Obstructive Lung Disease (GOLD) severity classification with stages 1–4 based on airflow obstruction^21^ in line with previous Markov models on the management of COPD.^22^ However, the GOLD stage 4 health state was not made available for undiagnosed patients as virtually no patients were newly identified as severe as GOLD stage 4 in previous case-finding studies.^11 12^ The model had a time cycle of 3 months; short enough to capture important COPD-related events such as exacerbations.^23^ The time horizon was 50 years assuming a maximum age of 100 years.

The base case starting cohort of patients was distributed across five of the thirteen health states, in line with the patient distribution observed in the TargetCOPD trial for the 50-year-old age group, where 52.7% were male (table 1).^12^ A percentage of 43.0% had no respiratory symptoms, 48.2% had symptoms but no airflow obstruction, and the remaining 8.8% were new COPD cases that were undiagnosed prior to participating in the trial. Among these newly diagnosed patients, 69.0%, 27.4% and 3.6% had COPD GOLD stages 1, 2 and 3, respectively.

**Table 1:** General model parameters related to case-finding processes

Parameter	Value	α	β
Starting cohort characteristics (percentage)*^12^			
Male	52.7	5999	5394
Asymptomatic without COPD	43.0	364	482
Symptomatic without COPD	48.2	364	482
Undiagnosed COPD	8.8	74	772
Proportion in GOLD stage 1	69.0	58	26
Proportion in GOLD stage 2	27.4	23	61
Proportion in GOLD stage 3	3.6	3	81
Natural history of development of COPD (percentage per year)			
Development of symptoms†^46 47^	2.0	135	6775
Incidence of COPD*‡ 48	.6	55	9945
Proportion of incident cases in GOLD stage 1§^49^	72.2	44	17
Proportion of incident cases in GOLD stage 2§^49^	27.8	17	44
Routine practice (percentage)^12^			
Probability of being diagnosed with COPD	0.8	337	41 692
Treatment after COPD diagnosis	29.3	3972	9585
Systematic case-finding activities (percentage)^12^			
Received questionnaire	99.9	12 175	1
Responded to questionnaire	35.5	846	1572
Reported symptom on questionnaire among responders	56.4	482	364
Spirometry conducted in those reporting symptoms	66.1	559	287
Diagnosed with COPD in those attending spirometry	39.8	87	2331
Utility			
Asymptomatic without COPD^15^	0.8394	1522	291
Symptomatic without COPD^15^	0.7549	8817	2862
Costs (£)^12^	**Value**	**α**	**λ**
Postal questionnaire	4.01	99	39
Booking and conducting spirometry test	55.27	24	0.5

Beta distribution: the symbols α and β are parameters that define a beta distribution, which is a continuous probability distribution bounded at the extremes by 0 and 1. The number of successes is α, while failure is β.

Gamma distribution: the symbols α and λ are parameters that define a gamma distribution, which is a continuous discrete distribution bounded at the extremes by 0 and ∞. The mean of the distribution is α(1/λ) and variance is α(1/λ).^2^

*Age -dependent parameters. Values presented are for individuals aged 50- year-olds.

†Based on clinical opinion, it was considered that incident cases account for 10% of prevalent cases (20%) of respiratory symptoms in the UK population, which was validated using values from Eagan (2002).

‡A longitudinal observational primary care database (Dutch Integrated Primary Care Information) follow-up study. The incidence rate was reported in 1000 person-years, which was then converted to a 1-year probability.

§Cohort study of Danish general population at years 0, 5 and 15 (Copenhagen City Heart Study). Of symptomatic normal at baseline that later developed COPD 15 years later, 72% and 28% had GOLD stages 1 and 2, respectively. This was assumed to be a fixed distribution.

**Figure 2:**
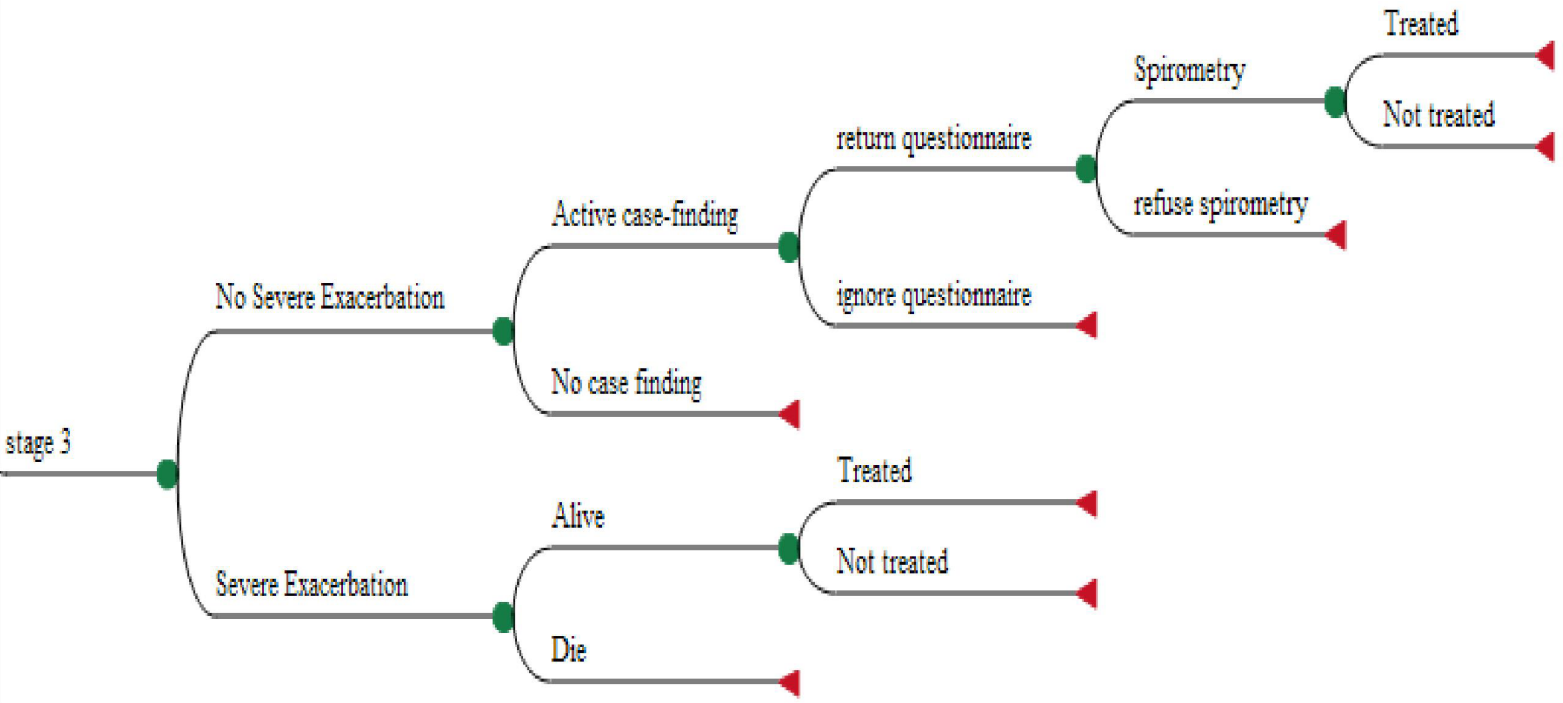
Example of pathway for an undiagnosed patient with GOLD stage 3 during a 3-month systematic case-finding cycle.

Transitions at every 3-month cycle were based on several assumptions to approximate the natural history and current management of COPD. Only patients who had developed symptoms could progress to any of the categories of undiagnosed COPD. Once a patient developed COPD, the model allowed movement to the immediate next worse GOLD stage. Direct deterioration beyond the next stage within a 3-month period was not allowed because COPD was assumed to progress slowly (e.g. movements from GOLD 1 directly to 3 and from GOLD 2 to 4 were not allowed). Transition from an undiagnosed to a diagnosed health state was permitted but not the reverse. Not all diagnosed patients received treatment (figure 2). Improvements were only permitted in treated patients. Undiagnosed GOLD stage health states were assumed to have the same baseline transitions to worse undiagnosed GOLD stages as diagnosed health states. Finally, there was a risk of exacerbation and death in a 3-month time cycle within any health state. The case-finding processes were modelled as events within each health state (figure 1). Systematic case-finding only occurred every 3 years, although a new diagnosis of COPD could arise through routine care in either strategy in every cycle.

### Data values used in the model

Most of the data related to the process of case-finding and diagnosis of COPD were derived from the active arm of the TargetCOPD trial^12^ and the associated Birmingham COPD cohort study^15^ (table 1 and table 2). Transition probabilities between GOLD stages were obtained from The Health Improvement Network (THIN) database, which holds longitudinal primary care information on over 11 million UK patients, including about 2 million with diagnosed COPD^24^ (see supplementary material for detailed estimation methods).

**Table 2:** Model parameters related to disease progression and outcomes (per annum)

	GOLD 1	GOLD 2	GOLD 3	GOLD 4	Dead
Transitions (probability)*^24^					
GOLD 1	0.9047	0.0876	0.0000	0.0000	0.0077
GOLD 2	0.0510	0.9001	0.0362	0.0000	0.0128
GOLD 3	0.0000	0.1044	0.8368	0.0324	0.0265
GOLD 4	0.0000	0.0000	0.0936	0.8187	0.0877
Transition for symptomatic patients*					
Symptoms, no COPD^48 49^	0.0040	0.0015	0.0000	0.0000	0.0026†
Exacerbation (probability)					
Severe exacerbation‡^15^	0.0270	0.0760	0.2720	0.3480	–
Mortality after severe exacerbation^33^	0.0703	0.0703	0.0703	0.0703	–
Treatment effect (OR)					
All-cause mortality^29^	0.9800	0.9800	0.9800	0.9800	–
Severe exacerbation^29^	0.8500	0.8500	0.8500	0.8500	–
Progression to the next GOLD stage§	0.8500	0.8500	0.8500	0.8500	–
Costs (£)¶					
Scheduled GP and hospital visits^19^	164.56	267.06	394.01	541.06	–
Inhaled medication^33^	485.16	567.84	735.96	824.52	–
Inpatient stay due to exacerbation^33^	2263.00	2263.00	2263.00	2263.00	–
Health outcomes					
Utility‡^15^	0.7197	0.7013	0.6798	0.5855	–
Disutility from severe exacerbation‡^15^	−0.2398	−0.2337	−0.2265	−0.1951	–
Utility gained from treatment^50^	0.0367	0.0367	0.0367	0.0367	–

*Age -dependent parameters. Values presented are for individuals aged 50- years-old.

†Value represents mortality risk in the general population.

‡Birmingham COPD cohort: data from the Birmingham Lung Improvement StudieS: an ongoing series of studies aimed at evaluating better strategies for identifying and managing COPD in primary care.^15^ Disutility data shows utility loss over 1 year: 50% utility loss in the first month and 25% utility loss for the second and third month per cycle. The impact of exacerbations on quality of life is greater in patients with less severe disease who also tend to be younger.^51^

§Expert panel comprised consultant pulmonologists, epidemiologists and senior health economist. The panel was presented with results of prior scoping reviews on the effect of treatment on exacerbation, mortality and lung function, but there was no review transition between GOLD stages. Given that the OR in reviews were around 0.85, the panel agreed then that the odds of treatment slowing disease progression to the next worse GOLD stage should be 0.85 for the base case.

¶Cost method was adapted and unit costs were updated to 2015 price year.

For pragmatic reasons, only severe exacerbations (i.e., those requiring inpatient stay^25^ were considered in this evaluation as these episodes alone account for over 84% of all COPD-related healthcare costs.^26^ The annual rate of severe exacerbations by undiagnosed and diagnosed GOLD stage was obtained from baseline data from the Birmingham COPD cohort.^15^ The rates were converted to quarterly transition probabilities and beta distributions were fitted about the point estimates.

Age-specific and sex-specific all-cause mortality rates were obtained from the life tables for England and Wales^27^ and applied to patients without COPD (online supplementary table S1). Rates were adjusted to avoid double counting COPD-related mortality. Age-specific all-cause mortality rates for diagnosed COPD patients were derived from the annual transition matrix generated from the THIN database (online supplementary table S7). COPD-adjusted all-cause mortality for the ‘disease free’ cohort was derived from the UK life tables (online supplementary table S1).

10.1136/thoraxjnl-2018-212148.supp1 Supplementary data

Prescription patterns in UK primary care show 29.6% of patients with COPD receive a long-acting beta-agonist (LABA)-based inhaled medication (excluding long-acting muscarinic antagonists (LAMA)), 9.5% receive a LAMA-based combination (excluding LABA), and 25.0% receive combinations that include LABA+LAMA.^28^ Treatment effects from published systematic reviews suggest reductions in risk of exacerbations of up to 27% (OR=0.73) for some dual inhaler combinations with further reductions for triple drug combinations.^9^ It was not practical to model treatment effects for each COPD inhaler combination on each type of outcome; therefore, a conservative simplifying assumption was made, using the point estimates from a meta-analysis of the effect of a single LAMA versus placebo on mortality (OR=0.98) and severe exacerbation (OR=0.85).^29^ The published evidence was largely based on patients with a FEV_1_ <60%, but the effect was assumed to be similar across all GOLD stages, although emerging evidence shows that patients with FEV_1_ >60% may have even greater capacity to benefit from early treatment.^30^

Only 29.3% of newly diagnosed patients were modelled to commence treatment annually.^28^ This annual rate was derived from a study that showed 82.7% of patients with COPD in the UK were on treatment 5 years post-diagnosis. This is likely to be a conservative estimate as reports from other countries suggest treatment initiation rates to be higher.^31^

Utility values for undiagnosed and diagnosed GOLD stages 1–4 health states were derived from baseline data from the Birmingham cohort,^15^ containing patients representative of a UK primary care COPD population in a stable condition and also symptomatic individuals without COPD. For individuals without symptoms, utility values were derived from a published age-adjusted algorithm, developed from utility values from the general population,^32^ as there was no utility value for ever-smokers in the general population in the literature. The model assumed that utility loss following severe exacerbation persisted for 3 months, in line with a previously published model.^33^ Disutility was modelled to be higher in the first month (50%) compared with the second (25%) and third (25%) month, after which quality of life was assumed to return to pre-exacerbation levels. This loss was applied to mean utility scores across all the four COPD severity levels.^34 35^

### Resource use and costs

The cost of systematic case-finding was estimated from the active arm of the TargetCOPD trial^12^ (tables 1 and 2, online supplementary table S3, table S4, table S5). Estimation of healthcare costs for the diagnosed and treated GOLD stages (table 2) followed existing costing frameworks.^33 35^ Cost of COPD-related inhaled pharmacotherapy was calculated using data from diagnosed patients in the Birmingham cohort. No cost was attached to routine care or comorbidities since these were assumed to be the same for both arms.

Unit costs were primarily from the Personal Social Services Research Unit,^36^ NHS reference costs and the British National Formulary.^37^ Costs were inflated to 2015 prices using the Hospital and Community Health Services inflation index^36^ where necessary.

### Assessment of cost-effectiveness

An incremental cost-effectiveness ratio (ICER) was calculated as a ratio of the mean difference in cost and the mean difference in QALY gained between systematic case-finding and routine practice and presented as cost per QALY gained. Discounting was applied to costs and outcomes at a rate of 3.5% in line with NICE guidance.^16^ Where available, data were entered into the model as distributions in order to fully incorporate the uncertainty around parameter values, so that a probabilistic sensitivity analysis could be undertaken. A gamma distribution was fitted for all cost parameters. A log-normal distribution, which accommodates the ratio nature of risk measures, was constructed for ORs. Beta distributions were fitted for all transition probabilities and utility estimates. The probabilistic sensitivity analysis was run with 10 000 simulations, and cost-effectiveness planes and cost-effectiveness acceptability curves (CEAC) were produced. The CEAC is the standard method for quantifying the likelihood that an intervention is more cost-effective compared with an alternative.

### Additional one-way sensitivity analyses

A series of one-way sensitivity analyses was conducted to assess how key parameters such as starting age of cohort, screening interval and time horizon affected the results. The impact of other important parameters such as questionnaire response rate, spirometry attendance rate, treatment initiation rates and the effectiveness of treatment with regards to exacerbations, mortality and quality of life gain were also explored.

## Results

The base case results for 50-year-old ever-smokers (table 3) showed that compared with routine practice, a 3-yearly systematic active case-finding strategy was more expensive but more effective, with a greater number of QALYs gained over a lifetime time horizon. The difference in cost was £466, with 0.0281 QALYs gained, producing an ICER of £16 596 per QALY gained.

**Table 3:** Base case result cost–utility analysis

Case-finding strategy	Mean values		Mean difference		ICER
	Cost (£)	QALYs	Cost (£)	QALY	(£/QALY)
Routine care	1007.64	14.1767			
Systematic case-finding	1473.51	14.2048	465.87	0.0281	16 596.28

ICER, incremental cost-effectiveness ratio; QALYs, quality-adjusted life-years.

**Figure 3:**
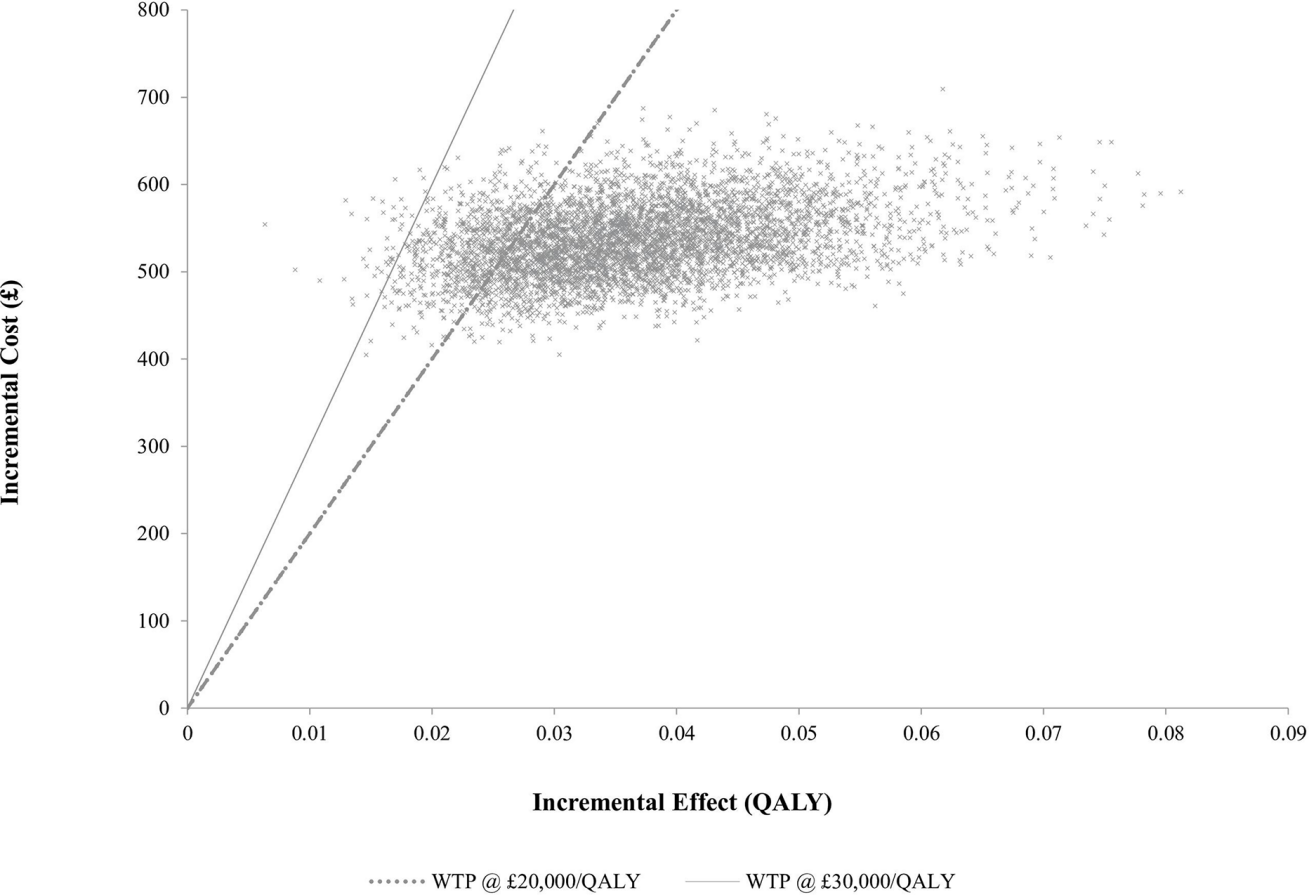
Cost-effectiveness plane for the comparison of systematic case-finding with routine care, based on 10 000 cost-effect pairs. QALY, quality-adjusted life-year.

Results from the probabilistic sensitivity analysis (figure 3) showed all 10 000 resampled points were clustered in the North-East quadrant, representing instances where systematic case-finding was more expensive and more effective than routine practice. A percentage of 78.4% of these points were below the £20 000/QALY willingness-to-pay threshold (WTP),^16^ which represents the probability of systematic case-finding being cost-effective at that threshold. The CEAC shows the probability of cost-effectiveness at different WTP thresholds (online supplementary figure S1)

### Sensitivity analysis

Varying the age for starting screening altered both the intervention costs and the QALYs gained (table 4). The most cost-effective age to begin screening in UK ever-smokers was estimated to be 60 years. Although the intervention costs were higher, the QALY gains from management of symptoms were also greater. Compared with younger age groups, a higher proportion of individuals aged 60 years had developed COPD during the first case-finding cycle, and therefore did not incur the costs associated with case-finding in subsequent cycles. The individuals aged 60 years were also young enough to maximally benefit from treatment of their symptoms relative to older cohorts.

**Table 4:** Sensitivity analysis results

	Cost difference	QALY difference	ICER (£/QALY)
Cohort age (years)			
40	356.32	0.0184	19 373.50
**50**	**465.87**	**0.0281**	16 596.28
60	520.27	0.0333	15 645.62
70	448.61	0.0265	16 915.53
Screening interval (years)			
1	910.08	0.0465	19 586.35
**3**	**465.87**	**0.0281**	16 596.28
5	334.09	0.0210	15 922.52
10	217.00	0.0143	15 219.88
Time horizon (years)			
20	316.94	0.0147	21 522.47
30	411.87	0.0226	18 206.16
40	458.44	0.0272	16 883.96
**50**	**465.87**	**0.0281**	16 596.28
Spirometry attendance rate			
10.5% (threshold 2)	159.21	0.0054	29 556.13
26.3% (threshold 1)	260.33	0.0130	20 097.49
**66.1% (base case**)	**465.87**	**0.0281**	16 596.28
Questionnaire response rate			
4.0% (threshold 2)	122.88	0.0040	30 364.90
11.6% (threshold 1)	219.19	0.0109	20 056.90
**35.0% (base case**)	**465.98**	**0.0281**	16 595.80
Utility gain from treatment			
0.0000	465.87	0.0115	40 456.80
0.0092 (threshold 2)	465.87	0.0155	30 011.41
0.0269 (threshold 1)	465.87	0.0233	19 999.67
**0.0367 (base case**)	**465.87**	**0.0281**	16 596.28

Threshold 1=willingness-to-pay threshold at £20 000 per QALY.

Threshold 2=willingness-to-pay threshold at £30 000 per QALY.

Questionnaire response rate after the initial invite in the TargetCOPD trial=15% (2312/15 387).**^12^**

Questionnaire response rate after the first reminder in the TargetCOPD trial=25% (3936/15 387).**^12^**

Base case values are in bold fonts.

ICER, incremental cost-effectiveness ratio; QALY, quality-adjusted life-year.

Annual case-finding yielded the most benefit but was the most expensive strategy, while a screening interval of 10 years had the lowest ICER thereby making the preferred screening interval from a cost-effectiveness perspective. The sensitivity analysis results also showed that the minimum required screening questionnaire response rate was 12% for systematic case-finding to remain cost-effective at the £20 000 per QALY threshold. Similarly, systematic case-finding was only preferred to routine practice if more than 26% of those who were invited for spirometric confirmation attended the session.

**Figure 4:**
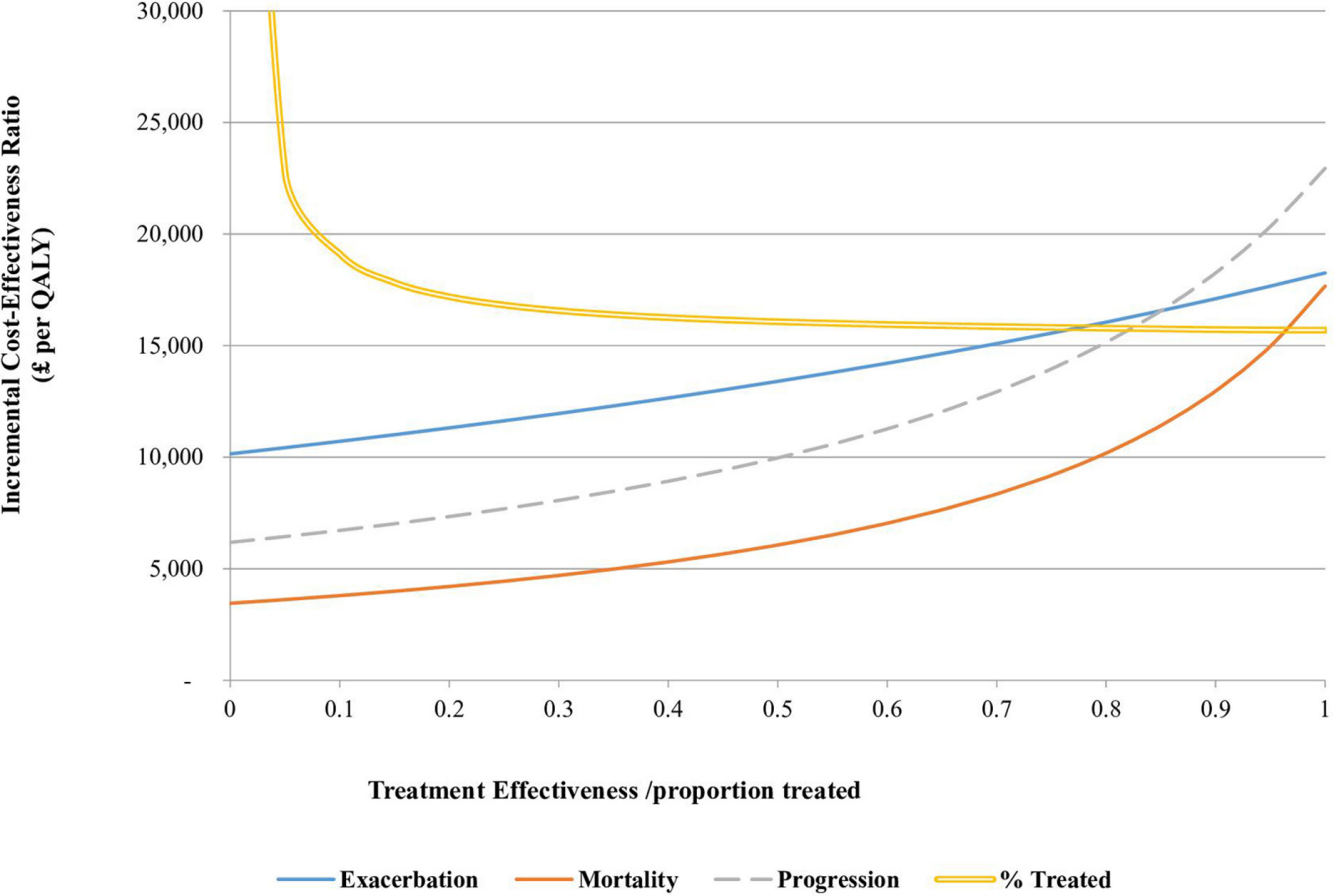
Multiple one-way sensitivity analyses showing the relationship between ICER and (1) the effect of treatment on exacerbation, (2) the effect of treatment on mortality, (3) the effect of treatment on disease progression, (4) the yearly treatment initiation rate in newly diagnosed patients. Treatment effectiveness estimates are expressed as ORs. ICER, incremental cost-effectiveness ratio; QALY, quality-adjusted life-year.

The model was also sensitive to the effectiveness of treatment on disease outcomes. The opportunity cost of systematic case-finding steadily increased as the effect of treatment worsened (figure 4). First, each variable was considered separately. When no impact on mortality was assumed, case-finding was still cost-effective at £17 663/QALY. No impact on exacerbations gave an ICER of £18 258/QALY. However, if no impact on progression (to worse GOLD stage) was assumed, the ICER rose to £22 943/QALY, and the threshold OR for cost-effectiveness at £20 000/QALY was 0.94. When the ORs for the effectiveness of treatment on all outcomes were simultaneously adjusted to 1, systematic case-finding was not preferred over routine practice (online supplementary figure S2), with an ICER of £28 811/QALY.

The model was also sensitive to the magnitude of the additional impact on quality of life, which was independent of the impact on quality of life and survival from progression, mortality and exacerbation (table 4). If the utility gain reduced to less than 0.0269, then systematic case-finding was no longer cost-effective at £20 000/QALY. Assuming treatment had no additional impact on quality of life resulted in an ICER of £40 457/QALY. Another important determinant of cost-effectiveness was the treatment initiation rate. A systematic case-finding programme was cost-effective as long as treatment was initiated in at least 8% of previously untreated patients yearly (figure 4).

## Discussion

There are as yet no published primary studies that provide data on the long-term cost-effectiveness of a systematic programme of case-finding for undiagnosed COPD. In their absence, this novel economic model aims to address this unanswered question using data from the best published sources available. We have shown that the systematic screening of ever-smokers aged 50 years and over, every 3 years is potentially a cost-effective strategy according to UK cost-effectiveness thresholds. The results were supported by the majority of the sensitivity analyses except in the most extreme scenarios. For case-finding to be cost-effective, a sufficient proportion of patients must respond to the initial screening questionnaire (12%) and attend the confirmatory spirometry test (26%). In our published trial, 15% responded after the initial invite without a reminder^12^ and more than 63% of those invited attended the spirometry test. Crucially, 1 in 12 (8%) of previously untreated patients must also be started on treatment yearly for systematic case-finding to remain cost-effective. Data from long-term follow-up for the TargetCOPD trial suggests that 12 months after diagnosis, 21% of case-found patients in the active case-finding arm were on the practice COPD Quality Outcomes Framework (QOF) register, suggesting they were likely to be receiving some treatment. Mean lifetime costs for both systematic case-finding and routine care are relatively low (less than £1500); however, this can be explained by the low incidence of COPD and a relatively low proportion of undiagnosed COPD in the starting cohort. Therefore, only a relatively small proportion of patients in the model will develop COPD over time and incur costs. Furthermore, in the case-finding strategy, as approximately only a third of patients respond to the questionnaire, only a small proportion will actually go onto receive spirometry and incur these additional costs.

We sought to explain why systematic case-finding was cost-effective despite the use of conservative assumptions, especially for treatment effectiveness. First, as our systematic case-finding approach was relatively inexpensive, only a small proportion of newly diagnosed patients needed to benefit from treatment for the intervention to be cost-effective. Second, once treatment commenced, the risk of exacerbation and mortality were simultaneously reduced. Fewer exacerbations result in lower loss in QALYs as well as cost savings from fewer admissions to hospital. Reduced risk of mortality among treated patients results in greater accumulation of QALYs compared with their untreated counterparts. Overall, mortality did not have a significant impact on the ICER because treated patients who survived longer also consumed more healthcare resources. There are also further benefits from the effect of treatment on disease progression, and we also assumed a small utility benefit of being on treatment independent of disease progression and exacerbations. If this additional benefit was removed, then case-finding was no longer cost-effective.

Ten yearly systematic active case-finding was the most cost-effective screening interval, although policymakers need to balance this against a greater proportion of the cohort remaining undiagnosed for longer and the value patients and practitioners place on early diagnosis.^38^

To the best of our knowledge, this is the first model to evaluate the long-term cost-effectiveness of a COPD case-finding strategy. The reliability of the main data sources that informed the model was a notable strength. Patient-level data from the TargetCOPD trial, the Birmingham COPD cohort and THIN dataset provided up-to-date information on both diagnosed and undiagnosed patients with COPD in primary care in the UK.

Another strength was the use of conservative estimates of the treatment effect to prevent overestimation of the benefits of systematic case-finding. The natural history of COPD in untreated patients remains largely unknown. Here, we assumed that untreated and treated patients had the same natural history. In reality, undiagnosed patients may have a slightly poorer quality of life from suboptimal management and the disease progression rate might be faster.^3 28^

This study, however, does have several limitations. The first limitation is the uncertainty around the effect of treatment on progression from one GOLD stage to the next.^39 40^ This estimate was not available in the literature. Although some previous studies have shown that treatment slows lung function decline (eg, changes in FEV_1_)^41 42^, there is currently no clear method for transforming changes in FEV1 decline into risk ratios that could be used in this model. Nonetheless, the reduced lung function decline in treated patients is an indication that treatment may reduce risk of progressing to a worse GOLD stage. However, in order to explore the uncertainty regarding the impact of treatment, extensive sensitive analyses were undertaken.

Additionally, the treatment effect as used in this model only captured the benefits associated with inhaled medications. Other interventions such as smoking cessation which has been shown to be effective in reducing COPD progression,^43^ pulmonary rehabilitation^8^ and self-management^35^ which improve HRQoL and reduce exacerbations, were not considered. Inclusion of other interventions would have made systematic case-finding more cost-effective but few patients receive these interventions, thereby making their wider benefit uncertain.

Another possible weakness is the use of the traditional GOLD staging criteria^44^ as airflow obstruction relates only weakly to quality of life. For instance, some patients with GOLD stage two may experience worse symptoms and impact than those with GOLD stage 3. Other symptom-based classification systems that are better predictors of prognosis now exist.^45^ However, there is no consensus regarding the most appropriate staging criteria, and the GOLD staging used here was the one used in previous literature that has informed inputs for assumptions used in the model.

We have also assumed that transitions between GOLD stages, exacerbation rates and utility values for undiagnosed states are the same as diagnosed (and untreated) GOLD stage health states. However, this assumption is supported by findings from cohort studies (eg, the CanCOLD study) that show that those with undiagnosed COPD have similar rates of health service use related to respiratory disease as those who have diagnosed COPD.^3^

Despite this, a further weakness lies in the assumption made regarding costs of undiagnosed disease. Only COPD-related costs are taken into account rather than all-cause costs. This may underestimate costs in the undiagnosed states, where there may be greater healthcare utilisation (eg, primary care visits) due to COPD, but the costs are not yet related to the condition. We also assume that untreated patients do not incur any healthcare cost until an admission for severe exacerbation occurs, whereas it is likely that some would have received prescriptions for their symptoms. However, it would be difficult to estimate these additional healthcare costs, and the conservative approach we have taken means that it is likely that case-finding would be more cost-effective with their inclusion.

A significant barrier to the implementation of case-finding programmes around the world has been the lack of evidence on whether the long-term benefit of early diagnosis and treatment outweighs the associated cost. Our economic model suggests that systematic case-finding leading to earlier diagnosis and treatment would provide benefits and value for money, despite uncertainty about treatment effectiveness in case-found patients and those with mild disease. The treatments would have to be almost completely ineffective on all important disease outcomes for regular case-finding to be a worse option than current practice. However, we recognise that this is not a primary study, and it would be strengthened by better knowledge about the natural history of the disease and treatment effectiveness. Ultimately, data from a case-finding trial with longer term health outcomes would provide more robust evidence. We have also provided information on potential starting age and screening intervals. The exact configuration of such case-finding activity may however depend on local factors such as competing pressures on national budgets. A further need is to explore more fully patient views on earlier diagnosis and the overall financial impact on primary healthcare organisations of a much larger population of COPD patients to manage. Should a new programme of case-finding be implemented, a clear pathway of care would need to be provided in order to ensure newly diagnosed patients are optimally treated, as current data suggest that this is seldom the case.^30^

## Conclusion

We conclude that a 3-yearly systematic approach to case-finding is likely to be cost-effective in the long term given the current management of patients with COPD in primary care setting. The true importance of early diagnosis and treatment of COPD will be better understood as more evidence emerges on the effect of treatment on COPD and the longer term results of case-finding trials are available. Longer term follow-up of newly diagnosed patients may also further clarify the natural history of COPD.
