# Supplementary material for: Model-based evaluation of the long-term cost-effectiveness of systematic case-finding for COPD in primary care
Source: Thorax. 2019 Jul 8;74(8):730–9. doi: 10.1136/thoraxjnl-2018-212148 (PMC6703126; doi:10.1136/thoraxjnl-2018-212148)
Supplement: Supplementary data [file thoraxjnl-2018-212148supp001.pdf]

Supplementary material

Table S1: COPD-adjusted all-cause mortality rates, by age and sex

Age	*Unadjusted mortality (%)*		*No deaths from all causes*		*No of death from COPD*		*% deaths from COPD*		*COPD adjusted mortality (%)*	
	Male m_x	*Female m_x*	*Male*	*Female*	*Male*	*Female*	*Male*	*Female*	*Male*	*Female*
40	0.1577	0.0883							0.1559	0.0876
41	0.1628	0.0958							0.1610	0.0950
42	0.1692	0.1058	3282	2125	37	18	1.127%	0.847%	0.1673	0.1049
43	0.1884	0.1157							0.1863	0.1147
44	0.2064	0.1271							0.2041	0.1260
45	0.2251	0.1383							0.2224	0.1364
46	0.2362	0.1447							0.2334	0.1427
47	0.2505	0.1623	5108	3286	61	45	1.194%	1.369%	0.2475	0.1601
48	0.2680	0.1711							0.2648	0.1688
49	0.2944	0.1926							0.2909	0.1900
50	0.3106	0.2159							0.3050	0.2110
51	0.3428	0.2347							0.3366	0.2294
52	0.3709	0.2561	7076	4856	127	110	1.795%	2.265%	0.3642	0.2503
53	0.4075	0.2784							0.4002	0.2721
54	0.4538	0.2981							0.4457	0.2913
55	0.4877	0.3408							0.4727	0.3277
56	0.5368	0.3681							0.5203	0.3540
57	0.5980	0.4042	9689	6501	298	249	3.076%	3.830%	0.5796	0.3887
58	0.6629	0.4394							0.6425	0.4226
59	0.7444	0.4783							0.7215	0.4600
60	0.8034	0.5239							0.7649	0.4898
61	0.8848	0.5825							0.8424	0.5446
62	0.9726	0.6303	14289	9677	685	629	4.794%	6.500%	0.9260	0.5893
63	1.0394	0.6778							0.9896	0.6337
64	1.1370	0.7383							1.0825	0.6903
65	1.2185	0.7968							1.1448	0.7371
66	1.3279	0.8616							1.2476	0.7970
67	1.4714	0.9686	21901	14938	1324	1120	6.045%	7.498%	1.3824	0.8960
68	1.6263	1.0806							1.5280	0.9996
69	1.8133	1.1788							1.7037	1.0904
70	1.9994	1.3208							1.8568	1.2100
71	2.2319	1.4533							2.0727	1.3313
72	2.5597	1.6612	26311	19268	1877	1617	7.134%	8.392%	2.3771	1.5218
73	2.7619	1.8449							2.5649	1.6901
74	3.0452	2.0417							2.8280	1.8704
75	3.3765	2.2788							3.1415	2.1080
76	3.7254	2.5435							3.4662	2.3528
77	4.1036	2.8630	34589	27917	2407	2093	6.959%	7.497%	3.8180	2.6484
78	4.6518	3.1770							4.3281	2.9388
79	5.0873	3.6497							4.7333	3.3761
80	5.7954	4.1666							5.4247	3.9050
81	6.5348	4.6844							6.1168	4.3903
82	7.4171	5.3068	41831	41029	2676	2576	6.397%	6.278%	6.9426	4.9736
83	8.3147	6.0756							7.7828	5.6941
84	9.3206	7.0031							8.7243	6.5634
85	10.5538	7.9108	40126	50909	2473	2413	6.163%	4.740%	9.9034	7.5358

86	11.8388	8.9452							11.1092	8.5212
87	13.2144	10.1286							12.4000	9.6485
88	14.7896	11.3019							13.8781	10.7662
89	16.5102	12.9950							15.4927	12.3791
90	18.1566	14.6089	25635	47921	1331	1462	5.192%	3.051%	17.2139	14.1632
91	20.1408	16.3333							19.0951	15.8350
92	23.2343	18.7654							22.0279	18.1929
93	25.0647	20.4129							23.7633	19.7901
94	26.2338	22.5065							24.8717	21.8199
95	29.7602	24.6122	7008	22856	243	383	3.467%	1.676%	28.7283	24.1998
96	33.3723	28.7120							32.2151	28.2309
97	36.4647	30.8803							35.2003	30.3628
98	39.6400	33.8692							38.2655	33.3017
99	42.0893	37.4876							40.6299	36.8594
100	43.2971	39.5028							41.7958	38.8408

Table S2 Active case finding processes and cost assumption per patient

Process	Assumption	Cost (£)
**Searches, screening**		
Running the search	20 minute of practice manager time per practice	0.01
Screening patient list	1 minute of GP time per patient selected	3.20
Attaching flags	15 seconds of administration time per patient record	0.94
Total		4.15
**Questionnaires**		
Drafting/approving letter	1 hour of practice manager time per practice per mail out	0.05
Administering mail-merge x 3	2 hours of administration time per practice per mail out	0.08
Administration the postal questionnaires	1.8 minutes of administration time per letter sent, plus stationery cost	0.67
Stationery cost for postal questionnaire	6 sheets of headed note paper & letter head, stamp, envelope & return envelope	1.74
Total / questionnaire		2.55
**Administrative**		
Completing the questionnaire in-clinic	6 sheets of headed notepaper & 1 minute of GP time per questionnaire	3.84
Processing questionnaires and appointment	30 minutes of administration time per questionnaire completed	11.23
**Appointment booking**		
Book appointments	10 minutes, stamp, letter and envelope for 100% and text message for 49% of appointments	4.32
cancelled or rebooked	All booked appointments divided by appointment attended	1.91
Total / appointment		8.26
**Spirometry**		
Staff costs	50 minutes clinical support worker time, 3 minutes of reception time	22.04
Training	4 weeks training, 2-day workshop, 1-day refresher course. Annual cost, assuming 372 tests per year	4.33
Room costs	50 minutes per appointment	12.55
Travel costs	Average 2.85 miles per attendance, 40p per mile	1.14
Equipment	Use of spirometer and laptop. Single use of mouthpiece, spacer and salbutamol.	10.87
Total		50.95

*Detailed micro-costing methods for the active-case finding has been published in an earlier paper**Error! Reference source not found.**

Table S3 Annual routine healthcare utilisation cost by severity

Healthcare	GOLD 1	GOLD 2	GOLD 3	GOLD 4	Unit cost (£)
Secondary care annual review (visits)	0.5	0.5	0.5	1.25	138.0 [41]
Primary care annual review (visits)	0.5	0.5	0.5	1.25	67.0 [41]
Secondary care spirometry (tests)	0.5	0.5	1	1	50.9 [52]
Primary care spirometry (tests)	0.5	0.5	1	1	18.0 [52]
Flu vaccination (treatment uptake rate)	1	1	1	1	6.6 [53]
Oxygen Therapy (days)	0	0	1.22	6.08	15.0 [52]
Prescriptions per annum (GP)	1	1	2	3	22.9 [42]
**Annual cost (£)**	**164.56**	**267.06**	**394.01**	**541.06**	
**Quarterly cost (£)**	**41.14**	**66.77**	**98.50**	**135.2**	

Table S4 Tariff and average cost of hospital admission for COPD exacerbation

HRG code	Description	Tariff (£)		Activities		Weighted cost (£)	
		2011	2015	Total	COPD	2011	2015
DZ21A	COPD or bronchitis with length of stay 1 day or less discharged home	475	499	72,547	62,390	187.12	196.58
DZ21B	COPD or bronchitis with intubation with major complications	3718	2,649	129	111	2.60	1.86
DZ21C	COPD or bronchitis with intubation with complications	2621	2,567	27	23	0.38	0.38
DZ21E	COPD or bronchitis with non-invasive ventilation without intubation with major complications	3684	3,696	3,777	3,248	75.56	75.80
DZ21F	COPD or bronchitis with non-invasive ventilation without intubation with complications	2678	2,909	1,106	951	16.08	17.47
DZ21G	COPD or bronchitis with non-invasive ventilation without intubation without complications	2168	1,637	246	212	2.90	2.19
DZ21H	COPD or bronchitis without non-invasive ventilation without intubation with major complications	3819	4,190	40,006	34,405	829.64	910.23
DZ21J	COPD or bronchitis without non-invasive ventilation without intubation with complications	2475	3,075	52,870	45,468	710.56	882.81
DZ21K	Chronic Obstructive Pulmonary Disease or Bronchitis without NIV without Intubation without complications	1849	2,442	13,448	11,565	135.02	178.33
Total				184156	158374.2	1959.87	2265.65

Table S5 Estimated cost of severe exacerbation

Resource use	% requiring resource	Unit cost (£)
Average cost of COPD hospital stay	0.80	2,265
Average cost of hospital at home program	0.20	1,803
Community nurse follow up	0.30	67
GP follow up (12 minute visit)*	0.30	45
Outpatient appointment follow up+	0.40	140
**Total**		2263.00

*Per hour of General Medical Service (GMS) activity
+Per hour of patient contact

Transition probabilities within stable state

The Health Improvement Network (THIN) database is a large primary care database containing patient-level data on over 550 general practitioners across the UK.

Table S6 Criteria for defining THIN cohort	
1	Aged 40 or older on or before April 1,2012
2	Confirmed diagnosis of COPD before April 1, 2012
3	Alive and actively registered to a general practice on or before April 1, 2012
4	Valid spirometry result within the previous 12 months before April 1, 2012

Demographic information and spirometry results on all registered COPD patients were extracted from the database. The extracted data was cleaned and a closed cohort of COPD patients was defined based on eligibility criteria in Table S6. Transitions between GOLD stages as well as mortality rates were computed after one year follow-up. In line with assumption that COPD is a slow progressive disease, worsening or improvement in lung function beyond the next health state was censored as shown in Table S7. Less than 1% of the THIN population was restricted by this assumption over the 12 months period. Given that the exact number of patients moving between the health states was known, compared to using reported data in the literature, a transition matrix was developed and a Dirichlet distribution was used to vary the probabilities in the sensitivity analysis.

Table S7 Annual transition matrix generated from the THIN database

		stage 1	stage 2	stage 3	stage 4	dead
GOLD 1	40-59	0.9047	0.0876	#	#	0.0077
	60-79	0.8565	0.1139	#	#	0.0296
	80-84	0.7864	0.0975	#	#	0.1161
GOLD 2	40-59	0.051	0.9001	0.0362	#	0.0128
	60-79	0.0487	0.8618	0.0438	#	0.0457
	80-84	0.0435	0.8009	0.0351	#	0.1205
GOLD 3	40-59	#	0.1044	0.8368	0.0324	0.0265
	60-79	#	0.0953	0.8035	0.0286	0.0727
	80-84	#	0.0828	0.705	0.0161	0.1961
GOLD 4	40-59	#	#	0.0936	0.8187	0.0877
	60-79	#	#	0.1198	0.7401	0.1401
	80-84	#	#	0.1756	0.6031	0.2214

Table S8 Probability of severe exacerbation and corresponding beta distribution by GOLD stage

Severity	Sample	Mean number per annum	Annual Probability	α	β	Source
GOLD 1	1034	0.0270	0.0266	28	1006	BLISS
GOLD 2	846	0.0760	0.0732	62	784	BLISS
GOLD 3	265	0.2720	0.2381	63	202	BLISS
GOLD 4	46	0.3480	0.2939	14	32	BLISS

Additional references:

52. Hertel N, Kotchie RW, Samyshkin Y, Radford M, Humphreys S, Jameson K. Cost-effectiveness of available treatment options for patients suffering from severe COPD in the UK: a fully incremental analysis. Int J Chron Obstruct Pulmon Dis 2012; **7**: 183-99.
53. Department of Health. The seasonal influenza immunisation programme Consultation: a review of the procurement of seasonal flu vaccine. 2011. https://assets.publishing.service.gov.uk/government/uploads/system/uploads/attachment_data/file/215577/dh_127231.pdf [Accessed 17/11/2016]

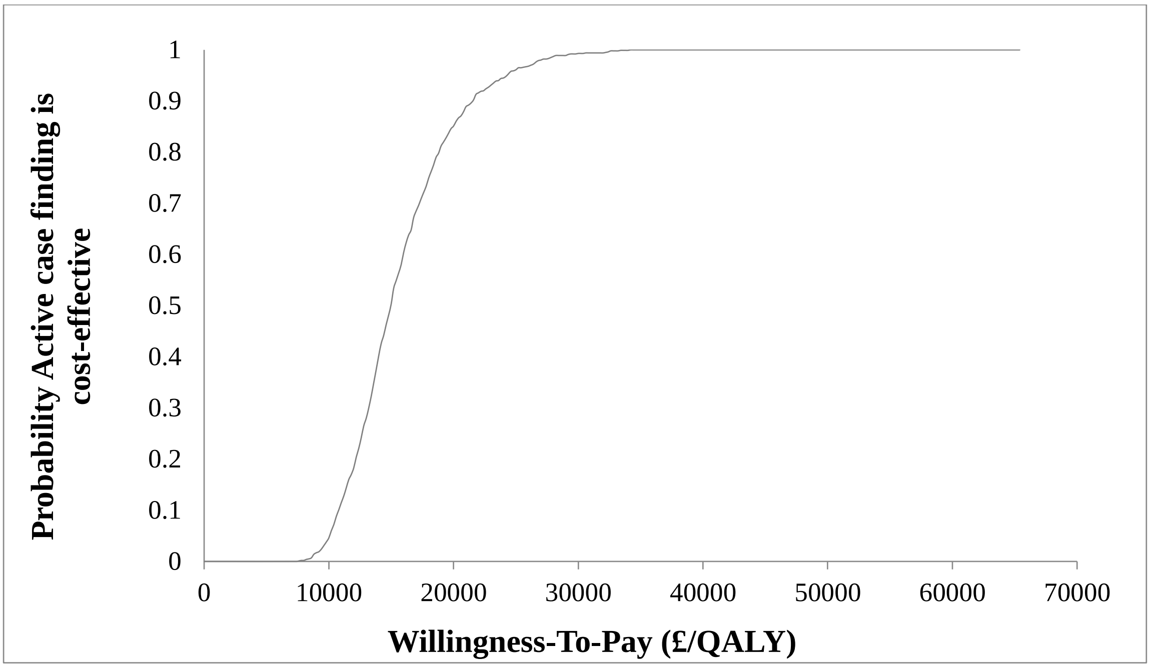

Figure S1: Cost-effectiveness acceptability curve comparing systematic case-finding is cost-effective with routine case-finding, based on 10,000 jointly resampled cost-QALY estimates

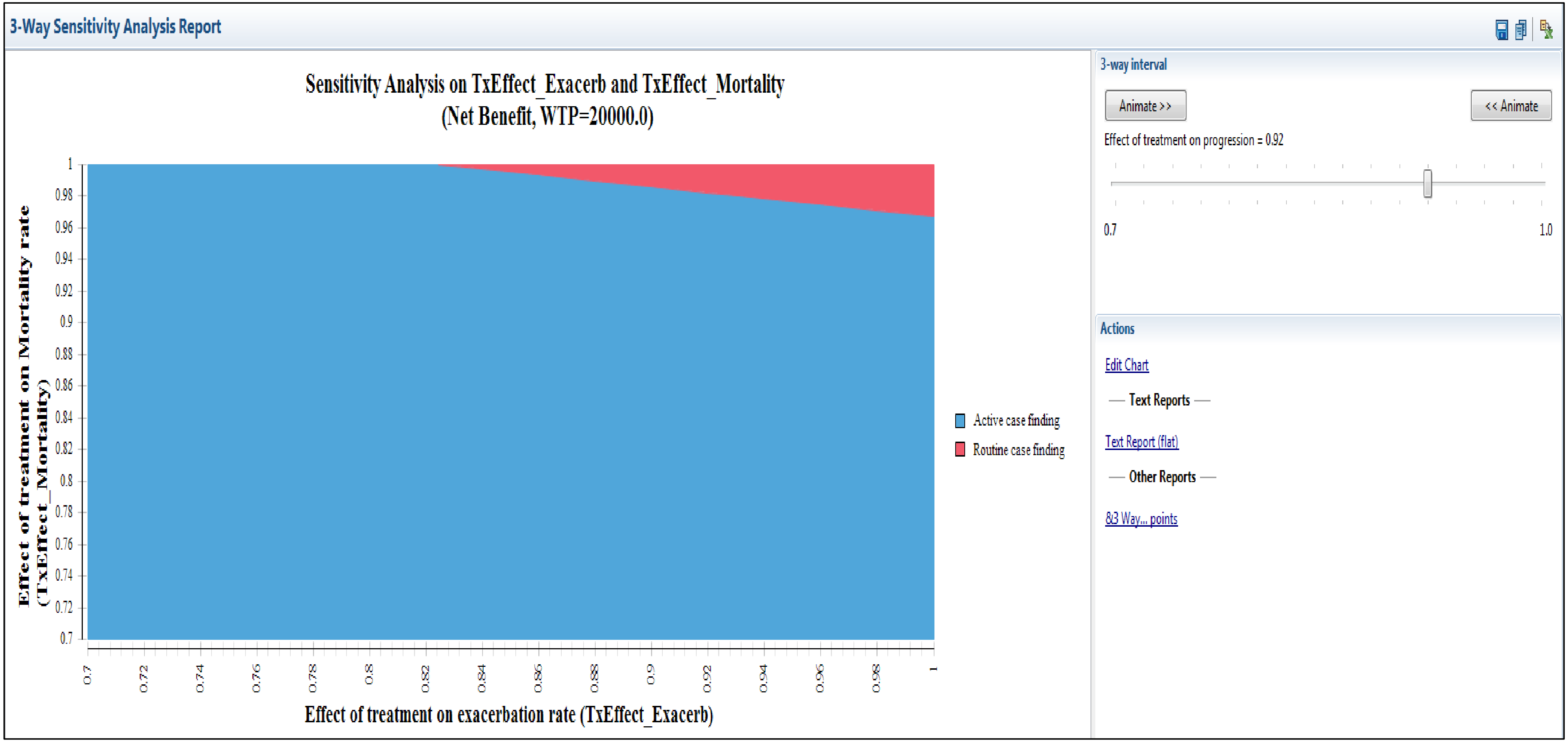

Figure S1: Animation snapshot of a three-way sensitivity analysis, showing the relationship between the effect of treatment on exacerbation (OR=0.7 to 1.0), mortality (OR=0.7 to 1.0) and disease progression (OR=0.92). The willingness-to-pay threshold was set at £20,000 per QALY. As the OR for the effect of treatment on disease progression shifts from 0.92 to 1.00, the probability that routine practice being cost-effective increases: The red area expands.
